# Case report: Secondary failure to tolvaptan in a patient with SCLC and paraneoplastic SIADH

**DOI:** 10.3389/fendo.2024.1382066

**Published:** 2024-05-13

**Authors:** Sheryl Menzi, Silvia Daniela Jaramillo, Stephan Pfister, Hubert Schefer, Andreas Werner Jehle

**Affiliations:** ^1^ Department of Internal Medicine, Hirslanden Klinik St. Anna, Lucerne, Switzerland; ^2^ Laboratory Medicine, University Hospital Basel, Basel, Switzerland; ^3^ Institute of Radiology and Nuclear Medicine, Hirslanden Klinik St. Anna, Lucerne, Switzerland; ^4^ Department of Oncology, Hirslanden Klinik St. Anna, Lucerne, Switzerland; ^5^ Transplantation Immunology and Nephrology, University Hospital Basel, Basel, Switzerland; ^6^ Department of Health Sciences and Medicine, University of Lucerne, Lucerne, Switzerland

**Keywords:** hyponatremia, SIADH, antidiuretic hormone, ADH, copeptin, SCLC, lung cancer, tolvaptan

## Abstract

The syndrome of inappropriate antidiuretic hormone secretion (SIADH) is frequent in lung cancer patients. Here, we report a case with persistent hyponatremia, which suggested malignant SIADH and facilitated an early diagnosis of small cell lung cancer (SCLC). A combined radio-chemotherapy led to a partial remission and resolution of SIADH. An early relapse was indicated by reoccurring severe hyponatremia and increased copeptin levels, which were used as surrogate markers for the antidiuretic hormone (ADH). As palliative immunochemotherapy, together with fluid restriction and solute substitution, were unable to control hyponatremia, treatment with the ADH V2-receptor antagonist tolvaptan was initiated. Over time, the dose of tolvaptan needed to be increased, paralleled by a well-documented exponential increase of copeptin levels. In summary and conclusion, this is a rare case of a secondary failure to tolvaptan with unique documentary evidence of increasing copeptin levels. This observation supports the hypothesis that exceedingly high ADH levels may lead to competitive displacement of tolvaptan from the V2 receptor.

## Introduction

Hyponatremia due to the syndrome of inappropriate ADH secretion (SIADH) is frequent in lung cancer patients (1). SIADH results from a variety of conditions such as pain, stress, infections, drugs, and CNS disorders, or it can be of paraneoplastic etiology. Malignant SIADH is caused by extra-hypothalamic production of the antidiuretic hormone (ADH), also called arginine vasopressin (AVP). Alternative causes of hyponatremia, such as adrenal insufficiency, need to be considered and essentially ruled out before SIADH and, more specifically, malignant SIADH can be diagnosed (2, 3).

The laboratory findings of SIADH include reduced serum sodium concentration (Na < 135 mmol/l) and low effective serum osmolality with an inappropriately high urine osmolality and continued renal sodium excretion, i.e., > 30 mmol/l (2, 4). Particularly in patients on diuretics, the fractional uric acid excretion with a cut-off of ≥ 12% has an excellent diagnostic value for the diagnosis of SIADH (5). Nonetheless, SIADH remains a diagnosis of exclusion, and low solute intake in relativity to water intake is an important differential diagnosis (2).

Copeptin, the c-terminal part of the precursor peptide of ADH, is a reliable surrogate marker for the less stable ADH (6). Copeptin has a limited value to differentiate between SIADH and other causes of hyponatremia, such as hypovolemic and hypervolemic hyponatremia (7), and mean copeptin values do not differ in SIADH of malignancy versus SIADH of other causes. Nevertheless, the highest measured values were found in malignant SIADH (8).

Tolvaptan is an orally effective ADH V2-receptor antagonist, which adds to the armamentarium to treat paraneoplastic SIADH, mainly if fluid restriction, salt tablets, urea, or hypertonic saline are ineffective or poorly tolerated. Considering the high price of this drug, it should be used judiciously. Tolvaptan binds competitively with 1.8 times greater affinity than ADH to the human V2-receptor (9), which results in decreased aquaporin-2 molecules in the apical membrane and thereby decreased permeability for water (9, 10). As a result, free water clearance is normalized and allows correction of hyponatremia.

In the present study, we report a case of a patient with paraneoplastic SIADH due to SCLC with secondary failure to tolvaptan paralleled by a sharp increase in copeptin levels.

## Case presentation

A 63-year-old woman was hospitalized in October 2019 with hyponatremia of 122 mmol/l. She was diagnosed with migraine, and the hyponatremia without a thorough workup was interpreted as pain-induced SIADH. Fluid restriction raised the serum sodium concentration to 130 mmol/l, and the patient was discharged with a fixed dose of flunarizine to treat her migraine and supplementary rizatriptan for acute attacks.

**Figure 1:**
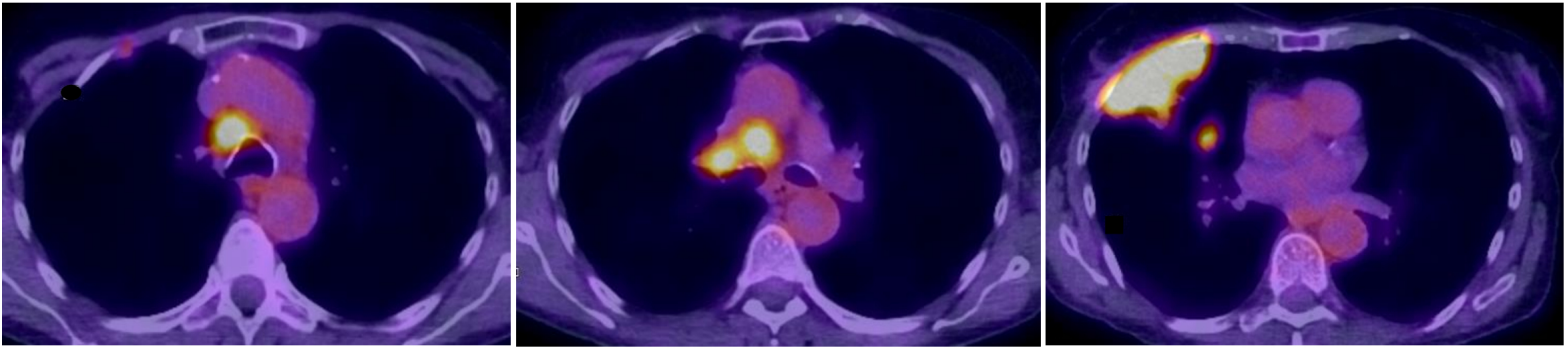
Fusion FDG-PET/CT axial slices with a large lesion in the upper lobe infiltrating the thoracic wall with a small lung metastasis and pathologic high FDG-uptake in mediastinal, hilar lymph nodes.

Two months later, in December 2019, the patient was readmitted with severe hyponatremia of 113 mmol/l. She suffered from headaches, nausea, and vertigo. Her serum osmolality was 236 mOsm/kg with a urine osmolality of 464 mOsm/kg and urine sodium < 20 mmol/l, which supported the clinical diagnosis of hypovolemia. After the attempt to correct hypovolemia with chicken soup and bouillon, the urine sodium concentration increased to 113 mmol/l, but serum sodium remained low at 120 mmol/l. Together with a high fractional excretion of uric acid of 32%, we diagnosed concomitant and persistent SIADH. In addition to fluid restriction, we initiated therapy with urea to facilitate free water clearance. Further, workup in this former smoker included a CT scan of the thorax (not shown) followed by a PET-CT scan (**Figure 1**), which revealed a pulmonary lesion in the right upper lobe. The biopsy obtained from the tumor resulted in the diagnosis of limited disease SCLC.

**Figure 2:**
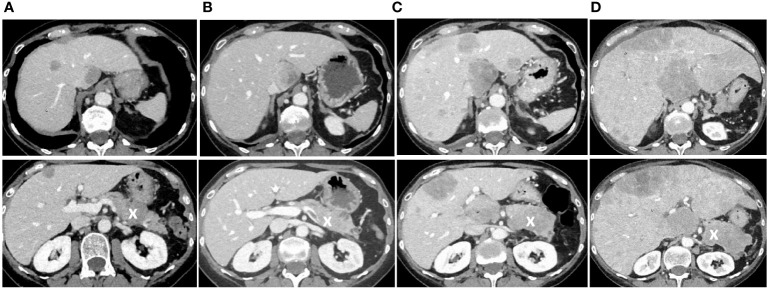
Follow-up CT scans showing tumor progression with hepatic and pancreatic metastases. **(A)** Follow-up CT scan three months after the end of the initial chemotherapy showing new liver metastases and a new lesion in the pancreas, marked with an X. **(B)** Follow-up CT scan seven months after the end of initial chemotherapy at the time when increasing doses of tolvaptan were necessary to control hyponatremia. **(C)** CT scan nine months after the end of chemotherapy at a time when tolvaptan was not effective anymore, and one month later **(D)** when the patient had beginning signs of liver failure and died shortly after that. In all images, the pancreas is marked with an X.

The patient received a combined radio-chemotherapy with cisplatin and etoposide between January and April 2020. During that period, she demonstrated a good partial remission, and her sodium levels remained between 130 and 141 mmol/l from March to June 2020. After that, the patient showed a drop of her serum sodium concentration to a minimum of 113 mmol/l. A CT scan of the thorax and abdomen revealed new nodules in the lung (not shown), multiple lesions in the liver, and one lesion in the pancreas (**Figure 2A**). The cerebral MRI did not indicate cerebral metastases. We obtained a liver biopsy, and the pathological examination was consistent with SCLC. The patient received palliative immunochemotherapy with atezolizumab, carboplatin, and etoposide. With fluid restriction, bouillon, and urea, serum sodium concentration increased to 124 mmol/l.

**Figure 3:**
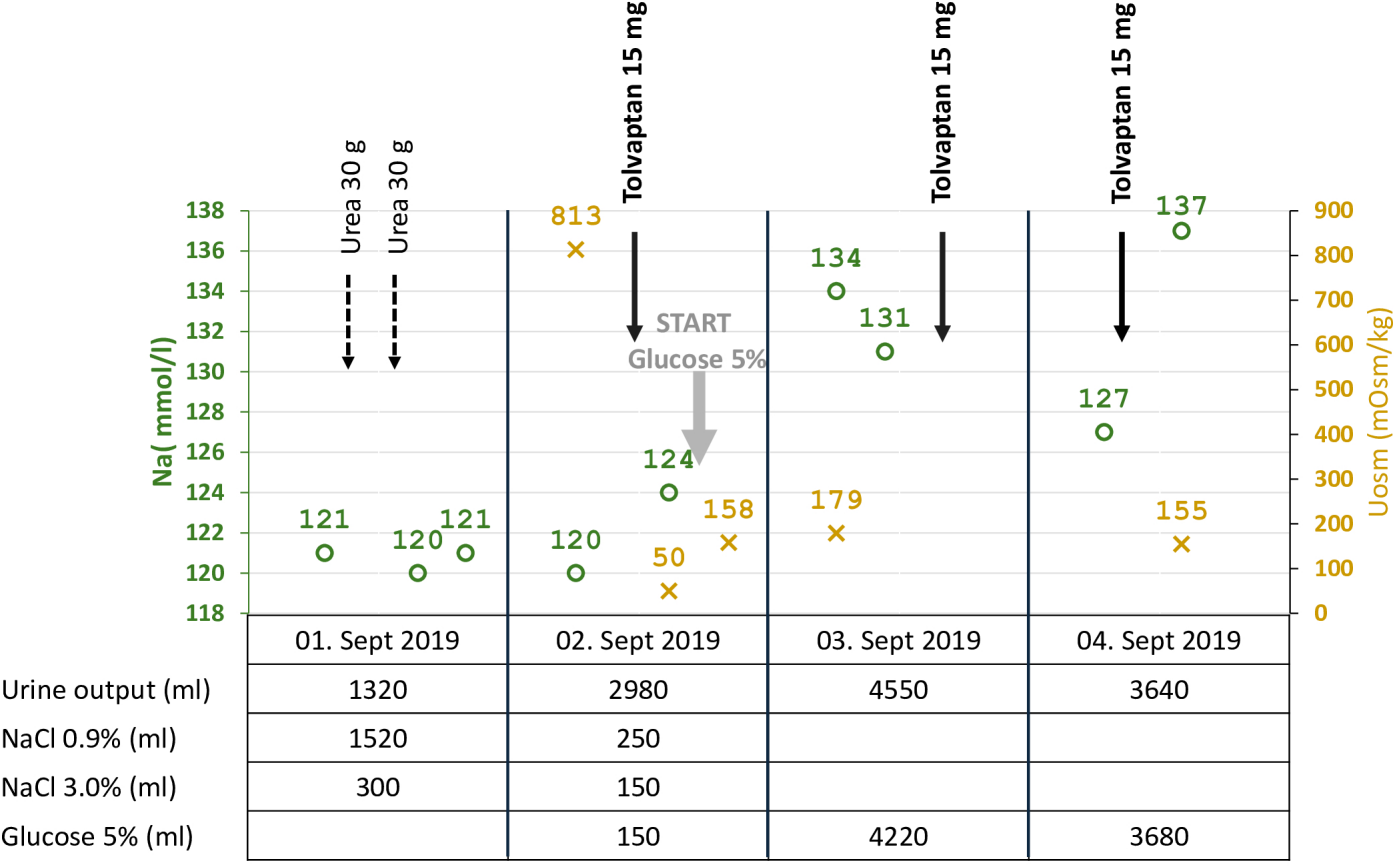
Start of tolvaptan: Serum sodium, urine osmolality, urine output, and volume/solute management. Summary of critical data before and after the start of tolvaptan. Within a few hours after the first dose of tolvaptan, the urine volume increased, paralleled by a drop in urine osmolarity from 813 to 50 mOsm/kg, and substitution of electrolyte-free water (glucose 5%) was started at a serum sodium concentration of 124 mmol/l to prevent too fast correction of hyponatremia.

**Figure 4:**
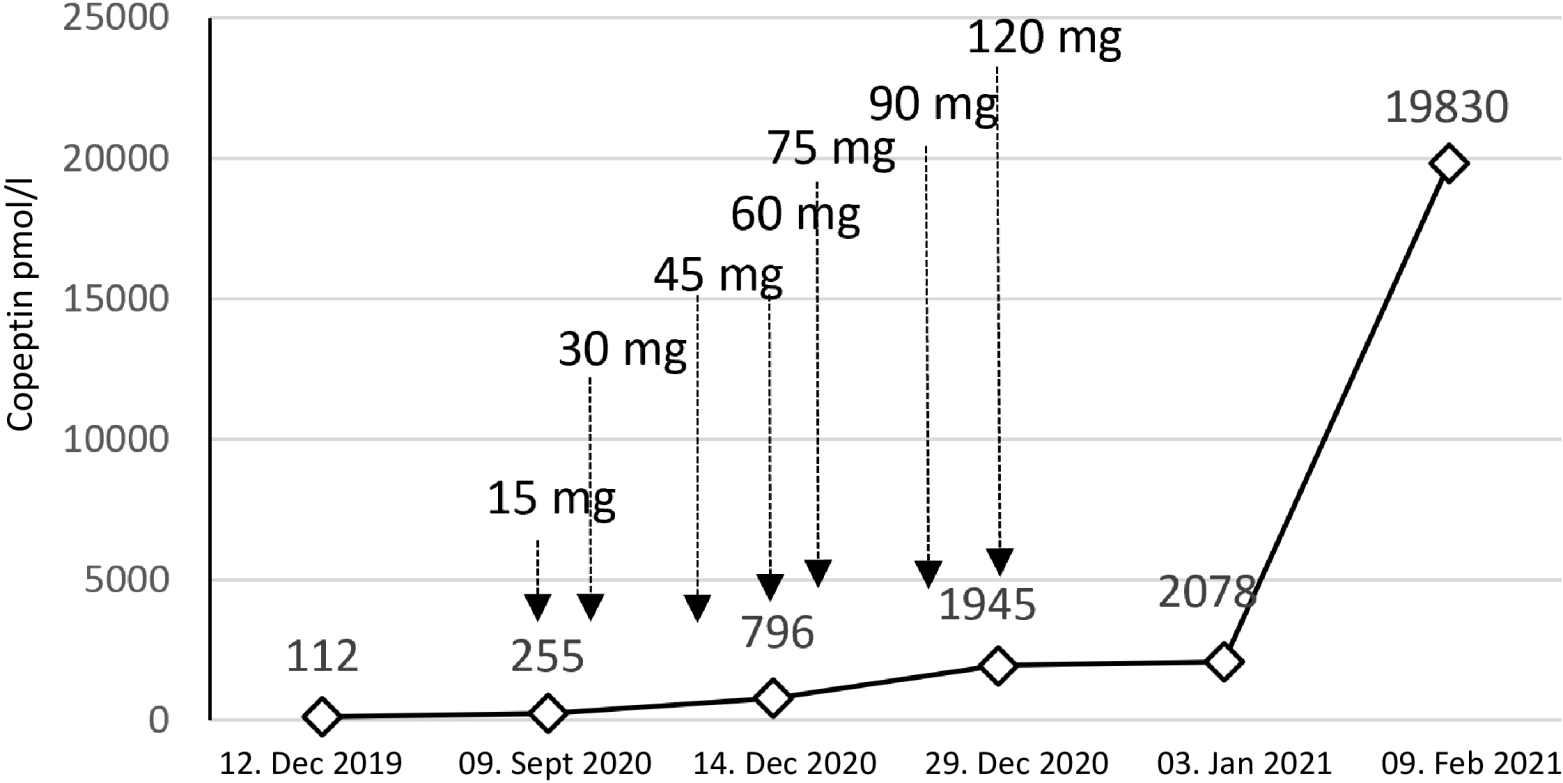
Copeptin values and tolvaptan doses. The figure shows the dramatic increase in copeptin over time. Treatment with tolvaptan was started with a dose of 15 mg per day. We needed to rapidly increase the dose to a maximum of 120 mg per day.

In September 2020, the patient had a urine osmolality of 813 mOsm/kg and did not respond sufficiently to urea and hypertonic saline solution (NaCl 3%) (**Figure 3**). Therefore, we initiated tolvaptan, starting with a dose of 15 mg. We observed a rapid increase in urine volume, and urine osmolality dropped to 50 mosmol/l within hours, requiring a volume substitution with electrolyte-free water, e.g. glucose 5%. Serum sodium increased from 124 mmol/l to 134 mmol/l within 24 hours (**Figure 3**). In the follow-up, increasing doses of tolvaptan - paralleled with a rapid increase of copeptin levels (**Figure 4**) - were necessary to control hyponatremia. To better interpret and classify the copeptin levels of our patient, we analyzed 932 copeptin concentrations measured in clinical routine of all patients from January 2019 to March 2021 (B·R·A·H·M·S Copeptin proAVP KRYPTOR assay). Of these values, 92% ranged between 0 and 50 pmol/l, 4.7% between 50 and 100 pmol/l, and 1.5% from 100 to 150 pmol/l, respectively. Five out of the 15 values above 150 pmol/l and the highest three values were measured in our patient.

During the further follow-up, the patient’s symptomatic hyponatremia with migraine attacks led to frequent hospitalizations. We registered a peak copeptin value of 19’830 pmol/l (**Figure 4**), and repeated CT scans showed further progression of the metastatic SCLC (**Figure 2B-D**). The patient died due to disseminated liver metastases and liver failure in February 2021.

## Discussion

In the case reported here, persistent hyponatremia suggested malignant SIADH and facilitated the diagnosis of SCLC. A combined radio-chemotherapy led to a partial remission and resolution of SIADH during a few months. An early relapse was indicated by reoccurring severe hyponatremia, after which we started therapy with tolvaptan at a time when palliative immunochemotherapy, together with fluid restriction and solute substitution, was unable to control hyponatremia. The initial response to 15 mg tolvaptan was strong, and the substitution of free water was necessary to prevent rapid correction of hyponatremia with the risk of osmotic demyelination (**Figure 3**). This suggests, that a starting dose of 7.5 mg tolvaptan may be preferred over 15 mg in the setting of chronic SIADH as the lower dose may be sufficient and the risk of a too rapid correction is diminished. Over time, the dose of tolvaptan needed to be increased, which can be explained by a well-documented exponential increase in copeptin levels (**Figure 4**). Although a strong correlation between ADH levels and tumor burden has not been observed consistently (11), repetitive CT scans in our patient (**Figure 2**) showed a link between increasing copeptin levels and tumor progression. Mechanistically, very high ADH levels may result in a competitive replacement of tolvaptan at the V2 receptor, as suggested in previous cases of two SCLC patients (12).

Following its approval in 2009, tolvaptan has been successfully applied in patients with SIADH due to SCLC. The first case reports were published in 2011 (13, 14). Amongst published cases, we identified four patients with SCLC and SIADH, where the initial effect of tolvaptan vanished (12, 15, 16). In two cases published in 2018 (3, 12), the patients showed an initial response to fluid restriction. Later, they were started on tolvaptan 7.5 mg daily with a good response. However, with cancer progression, tolvaptan with doses of up to 60 mg daily was not effective anymore. In the second patient, the copeptin value was 6088 pmol/l, i.e., extremely high as in the patient reported here. In 2019, a third case was reported (16) of a 48-year-old male with recurrent metastatic SCLC and hyponatremia of 107 mmol/l. Serum Na raised to 125 mmol/l with fluid restriction and NaCl 3%. He was then started on tolvaptan 15 mg daily, which was increased to 30 mg, then 60 mg, and finally to twice daily 60 mg without a response, and urine osmolality remained high at > 900 mOsm/kg. Tolvaptan treatment was discontinued, and serum sodium rose to 140 mmol/l with increasing doses of urea (up to 60 g per day), oral NaCl, and KCl combined with furosemide. A fourth case reported in 2022 (15) described a 61-year-old male with a locally advanced SCLC. He had symptomatic hyponatremia of 111 mmol/l. Symptoms subsided after receiving hypertonic saline, and sodium raised to 120 mmol/l. After that, he received tolvaptan 30 mg daily. During the next three months, he was readmitted multiple times with acute on chronic hyponatremia despite an increase of tolvaptan to 45 mg. Treating physicians then diagnosed disease progression with new liver metastases.

In summary, this case illustrates the importance of identifying underlying malignancy in a patient with persistent SIADH, as it can be the first manifestation of a malignant disease. When anticancer therapy and supportive measures fail to treat hyponatremia, a V2 receptor antagonist such as tolvaptan may enable the correction of hyponatremia, but this requires careful observation of the treatment response. This case demonstrates the rare observation of a secondary failure to tolvaptan associated with tumor progression and exponentially increasing copeptin levels, which supports the hypothesis of competitive displacement of tolvaptan from the V2 receptor. In conclusion, resistance to tolvaptan in malignant SIADH and a rise in copeptin levels, if measured, may be used as markers of tumor progression.

## Data availability statement

The original contributions presented in the study are included in the article/supplementary material. Further inquiries can be directed to the corresponding author.

## Ethics statement

Ethical approval was not required for the study involving humans in accordance with the local legislation and institutional requirements. Written informed consent to participate in this study was not required from the participants or the participants’ legal guardians/next of kin in accordance with the national legislation and the institutional requirements. Written informed consent was obtained from the individual(s) for the publication of any potentially identifiable images or data included in this article.

## Author contributions

SM: Writing – review & editing, Writing – original draft, Visualization, Validation, Investigation, Formal analysis, Data curation, Conceptualization. SJ: Writing – review & editing, Visualization, Validation, Methodology, Investigation, Formal analysis, Data curation. SP: Writing – review & editing, Visualization. HS: Writing – review & editing, Validation. AJ: Writing – review & editing, Writing – original draft, Visualization, Validation, Supervision, Project administration, Methodology, Investigation, Funding acquisition, Formal analysis, Data curation, Conceptualization.
